# Characteristics of preoperative atrial fibrillation in geriatric patients with hip fracture and construction of a clinical prediction model: a retrospective cohort study

**DOI:** 10.1186/s12877-023-03936-9

**Published:** 2023-05-18

**Authors:** Mingming Fu, Yaqian Zhang, Yuqi Zhao, Junfei Guo, Zhiyong Hou, Yingze Zhang, Zhiqian Wang

**Affiliations:** 1 Department of Geriatric Orthopedics, Third Hospital of Hebei Medical University, Shijiazhuang, Hebei People’s Republic of China; 2 Department of Orthopaedic Surgery, Third Hospital of Hebei Medical University, Shijiazhuang, Hebei 050051 People’s Republic of China; 3 NHC Key Laboratory of Intelligent Orthopeadic Equipment (Third Hospital of Hebei Medical University), Hebei, China; 4 Chinese Academy of Engineering, Beijing, 100088 People’s Republic of China

**Keywords:** Atrial fibrillation, Hip fracture, Preoperative period, The elderly, Prediction model

## Abstract

**Introduction:**

Atrial fibrillation is the most common atrial arrhythmia in the perioperative period and is associated with prolonged hospital stay, increased costs, and increased mortality. However, there are few data on the predictors and incidence of preoperative atrial fibrillation in hip fracture patients. Our aim was to identify predictors of preoperative atrial fibrillation and to propose a valid clinical prediction model.

**Methods:**

Predictor variables included demographic and clinical variables. LASSO regression analyzes were performed to identify predictors of preoperative atrial fibrillation, and models were constructed and presented as nomograms. Area under the curve, calibration curve, and decision curve analysis (DCA) were used to examine the discriminative power, calibration, and clinical efficacy of the predictive models. Bootstrapping was used for validation.

**Results:**

A total of 1415 elderly patients with hip fractures were analyzed. Overall, 7.1% of patients had preoperative atrial fibrillation, and they were at significant risk for thromboembolic events. Patients with preoperative AF had a significantly longer delay in surgery than those without preoperative atrial fibrillation (*p* < 0.05). Predictors for preoperative atrial fibrillation were hypertension (OR 1.784, 95% CI 1.136–2.802, *p* < 0.05), C-reactive protein at admission (OR 1.329, 95% CI 1.048–1.662, *p* < 0.05), systemic inflammatory response index at admission (OR 2.137, 95% CI, 1.678–2.721 *p* < 0.05), Age-Adjusted Charlson Comorbidity Index (OR 1.542, 95% CI 1.326–1.794, *p* < 0.05), low potassium(OR 2.538, 95% CI 1.623–3.968, *p* < 0.05), anemia(OR 1.542, 95% CI 1.326–1.794, *p* < 0.05). Good discrimination and calibration effect of the model was showed. Interval validation could still achieve the C-index value of 0.799. DCA demonstrated this nomogram has good clinical utility.

**Conclusion:**

This model has a good predictive effect on preoperative atrial fibrillation in elderly patients with hip fractures, which can help to better plan clinical evaluation.

## Introduction

The number of hip fractures worldwide is increasing due to an aging population. Surgery remains the treatment of choice for hip fractures to date [1, 2]. Atrial fibrillation is a common arrhythmia during perioperative period, with incidence rates ranged between 2%—60%, and it is related to poor outcomes [3–6].

There are many studies about atrial fibrillation associated with cardiothoracic surgery [7]. Underlying cardiac disease and direct surgical manipulation of the heart are thought to be associated with the development of perioperative atrial fibrillation. Hypertension and chronic obstructive pulmonary disease also identified as risk factors for perioperative atrial fibrillation associated with cardiothoracic surgery [8]. Orthopedic surgery is considered to have a relatively low perioperative incidence of atrial fibrillation compared with cardiothoracic surgery. Elderly and frail, functional limitations and comorbidities were patient characteristics in most hip fracture patients. So perioperative atrial fibrillation was common and considerable burdens still remained for hip fracture surgery [9].

In previous studies reported by Leibowitz D et.al [10], there was a highly correlation between perioperative atrial fibrillation and one-year mortality in elderly hip fracture patients, which was not attenuated by drug treatment of atrial fibrillation. Given the above, greater emphasis on prevention of perioperative atrial fibrillation might be warranted. Seong Jun Bae et.al [11] reported age and chronic obstructive pulmonary disease were predictors of postoperative atrial fibrillation in patients with hip fracture, which is one of the few studies. Whether these results apply to preoperative atrial fibrillation in hip fracture patients remains to be assessed. In addition, there are limited data on the characteristics of atrial fibrillation before hip fracture surgery.

At present, mechanisms and triggers of preoperative atrial fibrillation are still unclear in hip surgery. There is no gold standard risk score to predict individual risks of preoperative atrial fibrillation. We aimed to identify predictors of preoperative atrial fibrillation and propose a effective clinical prediction model.

## Materials and methods

### Study population

Electronic medical records were reviewed for all patients with hip fracture aged 65 years and over who underwent orthopedic repair surgeries from April 2017 to November 2020 in our department. Patients with multiple fractures, pathological fractures, malignant tumors, autoimmune disease, the history of chronic atrial fibrillation, time from injury to admission more than 1 week, and incomplete clinical data were not studied. This study obtained ethics approval from ethical review board of our hospital, and the informed consent was waived due to the retrospective design.

### Data collection and variables definition

The primary outcome was the new-onset atrial fibrillation before surgery. We compared characteristics of two groups and studied the predictors of preoperative atrial fibrillation. Baseline and perioperative variables related to preoperative atrial fibrillation were collected and analyzed. The following information was extracted from electronic medical records: gender, age, comorbidities, fracture type, perioperative complications, surgery delay time, hematological and biochemical indices at admission, left atrial size, and the information about preoperative atrial fibrillation.

Preoperative atrial fibrillation was defined as new-onset irregular cardiac rhythm without identifiable p waves on the ECG that was persistent and required medication. Cardiac arrhythmias can be detected by an electrocardiogram or ECG monitoring system.

### Statistical analysis

Differences between groups were compared by the Student's t-test or the Mann–Whitney U test for continuous variables. For categorical variables, the chi-square test or Fisher's exact test was used. The prediction model was developed by least absolute shrinkage and selection operator (LASSO) and multivariable logistic regression, and draw a nomogram. Area under the curve (AUC) of receiver operating characteristic curve (ROC) analysis was performed in order to assess how accurate the nomogram was at predicting risk. The calibration curve and decision curve analysis were used to assess calibration and clinical value of the nomogram separately. We used SPSS statistical software and R statistical software for our statistical analyses and graphics. The level of significance was set at *p* < 0.05.

## Results

### Characteristics of patients

From April 2017 to Sept 2019, a total of 2308 patients were screened to participate in this study. Among them, 893 patients were excluded and 1415 cases remained in the final analysis. Specifically, 138 patients had multiple fractures or pathological fractures; 281 patients received non-surgical treatment; 108 patients had chronic atrial fibrillation; 217 patients admitted with a delay of more than 1 week; 54 patients had malignant tumors and autoimmune disease, and 95 patients had incomplete data. Among analyzed patients, 101 (7.1%) occurred preoperative atrial fibrillation.

Table 1 summarizes characteristics of hip fracture patients. Most of the patients were women (71.4%) and mean age of patients was 79.6 years, with hypertension (46.6%) and cerebrovascular disease (41.0%) as the top two comorbidities. No differences in sex was found between groups, but a difference present in age, with preoperative atrial fibrillation patients being older than no-preoperative atrial fibrillation subjects (preoperative atrial fibrillation 82.3 ± 6.6 years, no-preoperative atrial fibrillation 79.4 ± 7.5 years,* p* < 0.001). The burden of chronic diseases, as assessed by Age-Adjusted Charlson Comorbidity Index (ACCI) was higher in patients with preoperative atrial fibrillation than without preoperative atrial fibrillation (5.3(1.3) versus 4.3(1.3), *p* < 0.001). The difference was statistically significant in the history of hypertension and coronary heart disease (*p* < 0.05). Although patients with preoperative atrial fibrillation seemed to have more diabetes and cerebrovascular disease, there was no significant difference. In addition to the above, C-reactive protein (CRP) and SIRI was found to be significantly higher in patients with preoperative atrial fibrillation.

**Table 1:** Baseline characteristics of geriatric patients with hip fracture

	**Total(*N* = 1415)**	**Preoperative atrial fibrillation(*N* = 101)**	**No-preoperative atrial fibrillation(*N* = 1314)**	***p* value**
Gender, *N* (%)				
Male	405 (28.6%)	35 (34.7%)	370(28.2%)	0.103
Female	1010 (71.4%)	66 (65.3%)	994(71.8%)	
Age, mean ± SD (years)	79.6 ± 7.5	82.3 ± 6.6	79.4 ± 7.5	< 0.001
Age group, *N* (%)				
< 80 years	670 (47.3%)	36(35.6%)	634(48.2%)	0.014
≥ 80 years	745 (52.7%)	65(64.4%)	680(51.8%)	
Mechanism of injury, *N* (%)				
Low energy	1375 (97.2%)	99 (98.0%)	1276 (97.1%)	0.825
High energy	40 (2.8%)	2(2.0%)	38(2.9%)	
Fracture types, *N* (%)				
Femoral neck fractures	642 (45.4%)	40 (39.6%)	602 (45.8%)	0.227
Intertrochanteric fractures	773 (54.6%)	61 (60.4%)	712 (54.2%)	
Comorbidities, *N* (%)				
Hypertension				
Yes	659 (46.6%)	61 (60.4%)	598 (45.5%)	0.004
No	756(53.4%)	40(39.6%)	716 (54.5%)	
Coronary heart disease				
Yes	402 (28.4%)	49 (48.5%)	363 (26.9%)	< 0.001
No	1013 (71.6%)	52 (51.5%)	961 (73.1%)	
Diabetes				
Yes	295 (20.8%)	26 (25.7%)	269 (20.5%)	0.209
No	1120 (79.2%)	75 (74.3%)	1045 (79.5%)	
Cerebrovascular disease				
Yes	580 (41.0%)	52 (51.5%)	528 (40.2%)	0.026
No	835(59.0%)	49 (48.5%)	786 (59.8%)	
ACCI	4.0(1.0)	5.3 ± 1.3	4.3 ± 1.3	< 0.001
Hematological and biochemical indices at admission				
CRP	27.7(43.6)	49.6(57.6)	26.9(40.8)	< 0.001
HB	113.2 ± 16.4	110.2 ± 14.3	113.5 ± 16.7	0.063
ALB	36.8 ± 4.5	37.2 ± 4.3	36.7 ± 4.5	0.346
SIRI	3.1(3.5)	5.6(2.0)	3.0(3.3)	< 0.001
Echocardiographic parameter				
Left atrial size	33.0 ± 4.6	32.5 ± 4.8	33.0 ± 4.6	0.298

Values are presented as mean ± standard deviation, median (interquartile range), or number (percentage) as appropriate

*SD* Standard deviation, *CRP* C-reactive protein, *HB* Hemoglobin, *ALB* Albumin, *SIRI* Systemic inflammatory responses index, *ACCI* Age-Adjusted Charlson Comorbidity Index

Among the 101 patients with preoperative atrial arrhythmia, the mean CHA2DS2‐VASc score was 4.5 ± 1.6 and 99 (98.1%) had CHA2DS2‐VASc score ≥ 2.

### Comparison of other preoperative complications that might induce preoperative atrial fibrillation and surgery delay time

The comparison of other preoperative complications that might induce preoperative atrial fibrillation and surgery delay time were shown in Table 2. In terms of type of complications, Patients with preoperative atrial fibrillation had a elevation in incidence of low potassium and anemia (*p* < 0.001). No differences were found in the incidence of heart failure between two groups. Moreover, surgery delay time of patients with no-preoperative atrial fibrillation was shorter compared to patients with preoperative atrial fibrillation (*p* < 0.001).

**Table 2:** Comparison of other preoperative complications that might induce preoperative atrial fibrillation and surgery delay time

Variables	Preoperative atrial fibrillation(*n* = 101)	No-preoperative atrial fibrillation(*n* = 1314)	*p* value
Heart failure			
Yes	28 (27.7%)	323 (24.6%)	0.481
No	73 (72.3%)	991 (75.4%)	
Low potassium			
Yes	43 (42.6%)	276 (21.0%)	< 0.001
No	58 (57.4%)	1038 (79.0%)	
Anemia			
Yes	54 (53.5%)	436 (33.2%)	< 0.001
No	47 (46.5%)	878(66.8%)	
Surgery delay time (days)	5.0(4.0)	4.0(3.0)	< 0.001

Values are presented as median (interquartile range), or number (percentage) as appropriate

### Selection of variables as predictors and derivation of the prediction model

**Fig. 1:**
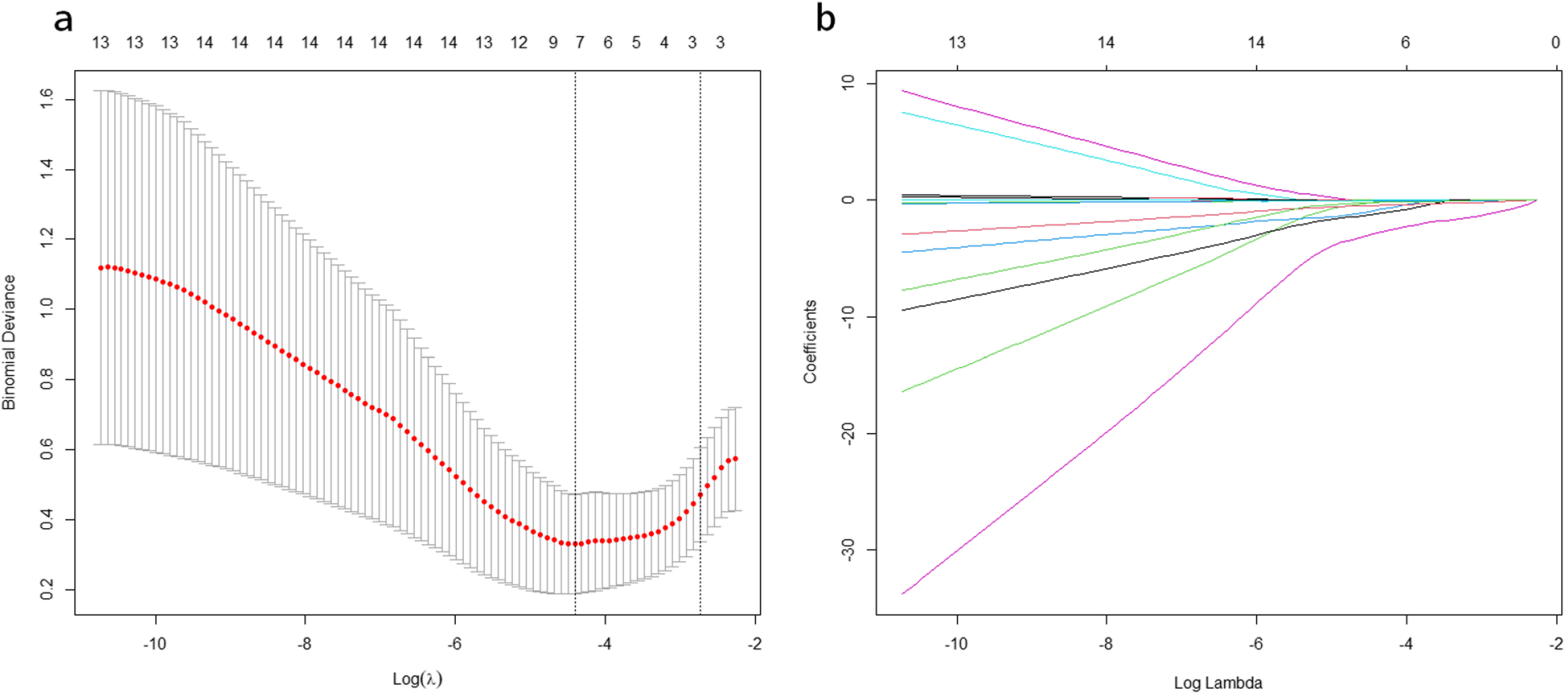
**a** LASSO regression of the variables. **b** Cross-validation for tuning the selection of parameters in the regression of LASSO

**Fig. 2:**
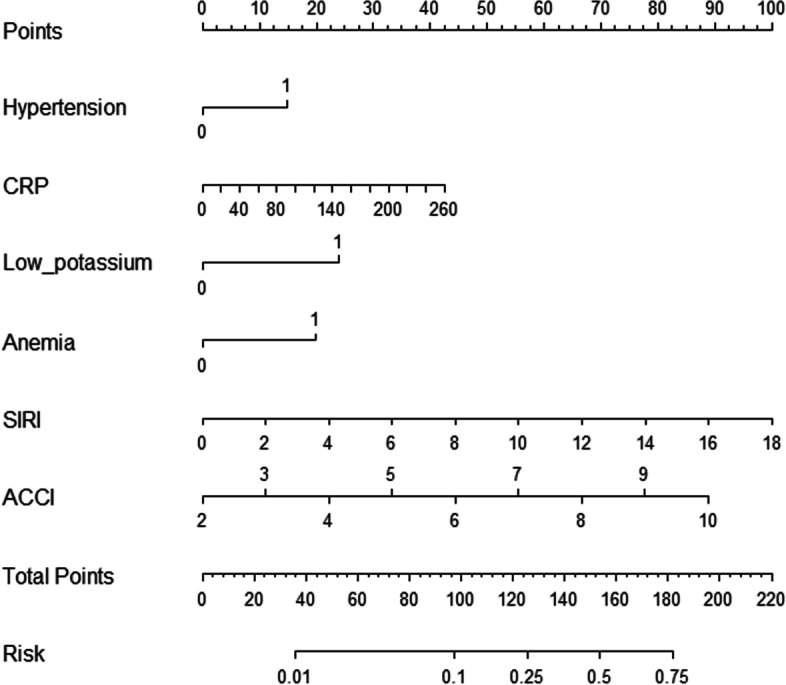
A model of risk prediction for preoperative atrial fibrillation. CRP, C-reactive protein; SIRI, systemic inflammation response index; ACCI, Age-Adjusted Charlson Comorbidity Index

With LASSO regression, 8 variables were selected. All 8 variables were then analyzed with the logistic regression model (shown in Fig. 1). There were 6 variables that remained in the model:hypertension (OR 1.784, 95% CI 1.136–2.802, *p* < 0.05), CRP at admission (OR 1.329, 95% CI 1.048–1.662, *p* < 0.05), SIRI at admission (OR 2.137, 95% CI,1.678–2.721 *p* < 0.05), ACCI (OR 1.542, 95% CI 1.326–1.794, *p* < 0.05), low potassium(OR 2.538, 95% CI 1.623–3.968, *p* < 0.05), anemia(OR 1.542, 95% CI 1.326–1.794, *p* < 0.05). Table 3 summarizes the findings of the analysis among hypertension,CRP at admission, SIRI at admission, ACCI, low potassium and anemia. By combining these independent predictors, a model was built and presented as a nomogram (shown in Fig. 2).

**Table 3:** Prediction factors of preoperative atrial fibrillation in geriatric patients with hip fracture

Intercept and variable	β	Odds ratio	95% CI	*p* value
Hypertension	0.579	1.784	(1.136, 2.802)	0.012
CRP at admission	0.006	1.329	(1.048, 1.662)	0.018
SIRI at admission	0.217	2.137	(1.678, 2.721)	< 0.001
ACCI	0.433	1.542	(1.326, 1.794)	< 0.001
Low potassium	0.931	2.538	(1.623, 3.968)	< 0.001
Anemia	0.772	1.542	(1.326, 1.794)	0.005

*CRP* C-reactive protein, *SIRI* systemic inflammatory responses index, *ACCI* Age-Adjusted Charlson Comorbidity Index

### Evaluation of the prediction model

**Fig. 3:**
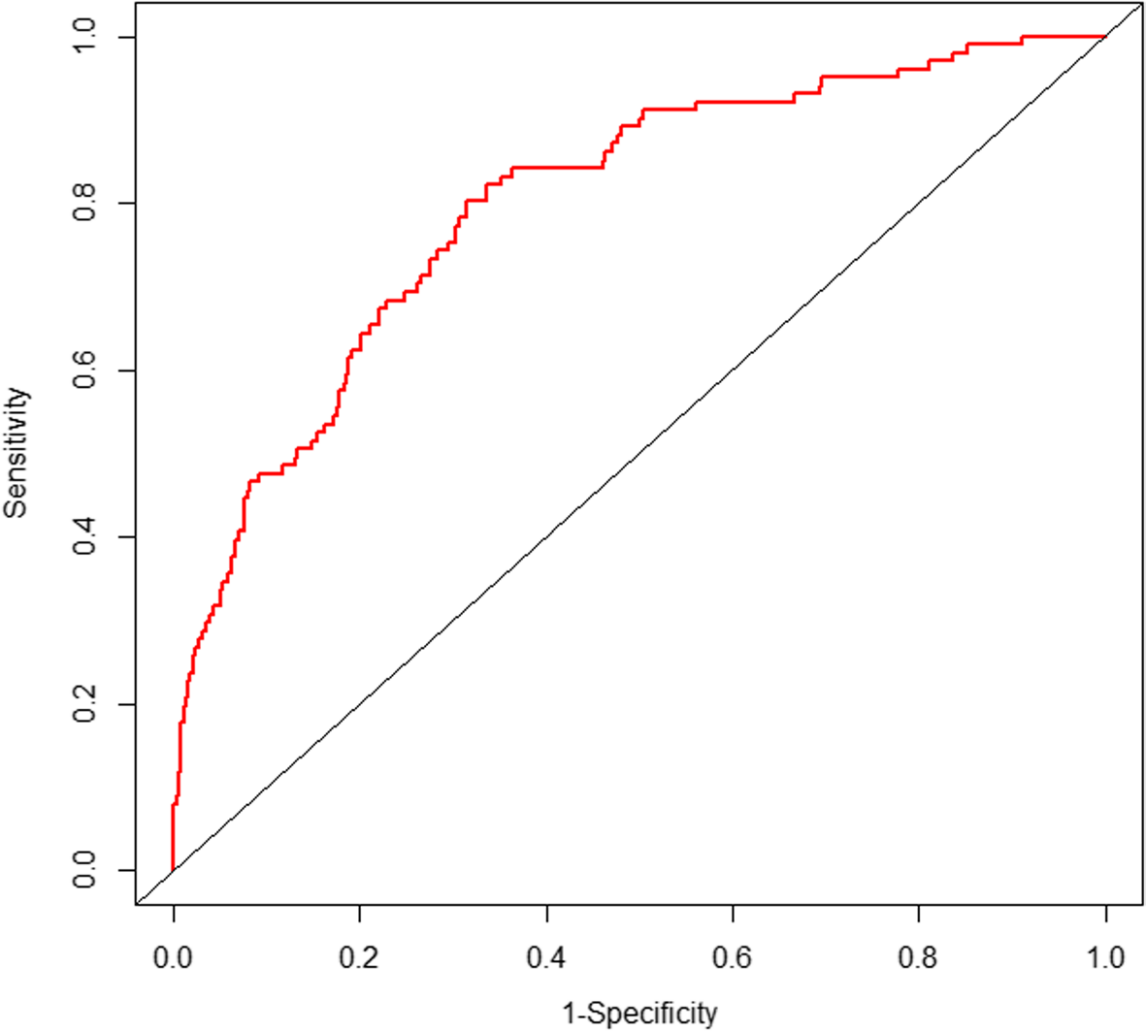
Analysis of ROC curve for the predictive values of preoperative atrial fibrillation

**Fig. 4:**
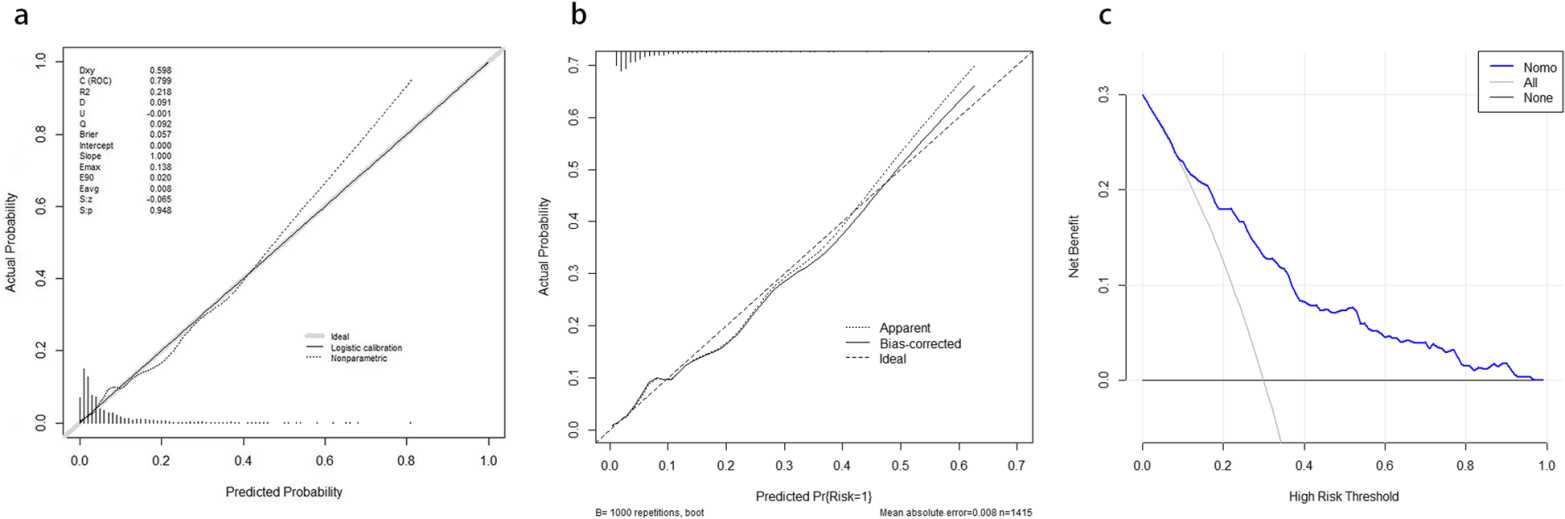
Calibration curves and Decision curve analysis of nomogram

Calibration curves used for estimating preoperative atrial fibrillation in hip fracture patients showed good agreement across the cohort.The AUC for the predictive nomogram model was 0.799(95% CI 0.754–0.844) (shown in Fig. 3). With a *p* value of 0.948, The calibration curve indicated a good calibration effect of the nomogram. The internal validation C-index using bootstrapping (resampling = 1000) is 0.799. DCA demonstrated this nomogram has good clinical utility (shown in Fig. 4).

## Discussion

Characteristics of preoperative atrial fibrillation in elderly patients with hip fracture was analyzed and a nomogram prediction was constructed in this study. In our results, the incidence of preoperative atrial fibrillation is 7.1%, and they had a considerable risk of thromboembolic events. Moreover, we found surgery delay time of patients with no-preoperative atrial fibrillation was shorter compared to patients with preoperative atrial fibrillation. Six prediction factors constitute the prediction model, including hypertension, CRP, SIRI, ACCI, low potassium and anemia, which are easily available in clinical practice. The nomogram could predict the preoperative atrial fibrillation risk in hip fracture patients accurately.

Nearly all elderly hip fracture patients with preoperative atrial fibrillation are at significant risk for thromboembolic events based on the CHA2DS2-VASc score. Previous research has shown that many episodes of atrial fibrillation are asymptomatic and may go undetected until a thromboembolic event or other serious complication occurs. Furthermore, hip fracture patients of our study are the elderly with a variety of comorbidities, and the presentation of illnesses in this population is often atypical, making clinical diagnosis difficult [12]. Therefore, the true incidence of atrial fibrillation in our study population may have been underestimated. Given the detrimental impact of AF on the prognosis of patients with hip fractures, prevention, timely diagnosis, and aggressive treatment are crucial. As with any preventable disease, predictors can be divided into modifiable and non-modifiable predictors. For modifiable predictors, identifying and avoiding its exposure is essential. However, high-quality research remains scarce, which is about the prediction model for preoperative atrial fibrillation on elderly hip fracture patients. Compared with no-preoperative atrial fibrillation, surgery delay time of patients with preoperative atrial fibrillation was significant longer. In addition to atrial fibrillation, the prolonged surgery delay time is likely related to overall condition of the patient. Left atrial dilatation is an important part of atrial adverse remodelling and is a strong predictor of atrial fibrillation. large left atrial volumes were associated with a high prevalence of persistent/permanent atrial fibrillation. In our study, there was no difference in left atrial size between the two groups.This is because most episodes of atrial fibrillation in our study were associated with perioperative factors, and most patients had only one episode without progression to persistent atrial fibrillation. Therefore, there was no significant change in left atrial volume. Of course, further studies on the mechanism of perioperative atrial fibrillation are needed to better explain this phenomenon.

Hypertension, CRP, SIRI, ACCI, low potassium and anemia were significant predictors of preoperative atrial fibrillation. The pathophysiology of the development of preoperative atrial fibrillation is not fully understood. Some studies reported that the pathogenesis of postoperative atrial fibrillation might be related to intraoperative sympathetic nerve activation, oxidative stress, systemic inflammatory response and complement activation [13]. We believed that this pathogenesis also applied to preoperative atrial fibrillation. It is common for elderly patients to suffer a severe traumatic stress response after hip fractures, including neuroendocrine and immunoinflammatory responses and changes in metabolic function [14–16]. As a result of stress, the sympathetic nervous system releases catecholamines and glucocorticoids, which cause sodium retention, vasoconstriction, and heart rate to increase and ultimately leading to perioperative myocardial ischemia and injury. On the other hand, immuno-inflammatory promoted production of inflammatory cytokines and superoxide, leading to perioperative myocardial injury. Changes in the metabolic function may lead to anemia, low albumin, low potassium and others.

Gregory M Marcus et al. discovered that atrial fibrillation was independently associated with inflammation, but the role of inflammation in the genesis, maintenance, or propagation of atrial fibrillation is poorly understood [17]. An indicator of the inflammatory response, CRP is used to assess the severity of traumatic stress [18–20]. Surgical trauma, fracture, infection and other factors affect the prognosis. The systemic inflammatory response index (SIRI) is based on the number of monocytes, lymphocytes and neutrophils in the peripheral blood and is used to describe the balance between the inflammatory response and the patient's immune status [21]. It is an affordable and accessible inflammatory marker. Previous research reported that SIRI might be related to the acute coronary syndrome [22]. In present study, we found CRP and SIRI were significant predictors of preoperative atrial fibrillation, that was to say inflammation was likely related to the genesis of preoperative atrial fibrillation.

Previous studies reported hypertension was related to atrial fibrillation [23–25]. In our study, hypertension was also one of significant predictors of preoperative atrial fibrillation in geriatric hip fracture patients. This was consistent with findings reported in previous studies. The mechanism might be associated with both electrical and structural atrial remodeling due to long-term elevated blood pressure. Activation of the renin–angiotensin–aldosterone system is the common pathophysiological basis of hypertension and atrial fibrillation. RAAS activity is increased in elderly patients with hip fracture and hypertension. As the main drug of the renin-angiotensin system, angiotensin II can increase intracellular free calcium ions, change potassium conductance, and induce the occurrence of transient inward current, thereby promoting the development of angiotensin II, which might contribute to the development of atrial fibrillation [26, 27].

Developed by Charlson et al., the Charlson Comorbidity Index is a measure of mortality risk resulting from comorbidities [28]. When age is taken into account as a correction variable, ACCI is used to evaluate prognosis, which is based on a calculation that incorporates both age and important underlying diseases [29–31]. In addition, ACCI demonstrated remarkable accuracy in predicting complications after surgery [30]. Studies showed that comorbidities were independently associated with atrial fibrillation, such as heart disease and chronic kidney disease etc. However, there was no report on the relationship between ACCI and preoperative atrial fibrillation in hip fracture patients. In our study, ACCI was one of significant predictors of preoperative atrial fibrillation in geriatric hip fracture patients. Age and coronary heart disease were risk factors of atrial fibrillation in previous studies. In contrast, we did not find a relationship between age and coronary heart disease and the risk of preoperative atrial fibrillation. Although there was a significant difference between the two groups in terms of age and coronary heart disease in univariate analysis, no significant difference was found in multivariate regression analysis.It was likely related to that ACCI had combined the effect of both age and coronary heart disease.

Elderly patients with hip fractures may also have other complications before or during the preoperative onset of atrial fibrillation, such as pulmonary infection, low albumin, hypokalemia, anemia, etc. Previous studies have reported that these complications may be predisposing factors for postoperative atrial fibrillation. Our results are similar to previous reports. Hypokalemia and anemia were significant predictors of preoperative atrial fibrillation. Anemia excited sympathetic nerves to induce atrial fibrillation. Low potassium causes cellular hyperpolarization, elevated resting potential, and increased cellular excitability, increasing the risk of arrhythmia.

This study has several limitations. First, it is a single-center, retrospective cohort study, which has an inherent limitation. Second, whether patients accept hospitalization is affected by many factors, including the economic level of the patient, and the number of beds of department, which may cause selection bias. Third, affected by incidence, the population is relatively small. Although we used clinical diagnoses as labels, the diagnoses of atrial fibrillation themselves may be a bit biased due to suspicious medical histories. Although nomogram had been extensively tested in self-initiated in-house validation testing, but further studies on multiple patients and external data from multiple locations are required to further confirm the results.

## Data Availability

Data are available from the authors upon reasonable request and with permission of corresponding author.
